# Instantaneous Inactivation of Herpes Simplex Virus by Silicon Nitride Bioceramics

**DOI:** 10.3390/ijms241612657

**Published:** 2023-08-10

**Authors:** Giuseppe Pezzotti, Eriko Ohgitani, Saki Ikegami, Masaharu Shin-Ya, Tetsuya Adachi, Toshiro Yamamoto, Narisato Kanamura, Elia Marin, Wenliang Zhu, Kazu Okuma, Osam Mazda

**Affiliations:** 1 Ceramic Physics Laboratory, Kyoto Institute of Technology, Sakyo-ku, Matsugasaki, Kyoto 606-8585, Japan; b0151006@edu.kit.ac.jp (S.I.); wlzhu@kit.ac.jp (W.Z.); 2 Department of Molecular Genetics, Institute of Biomedical Science, Kansai Medical University, 2-5-1 Shinmachi, Hirakata 573-1010, Japan; 3 Department of Immunology, Graduate School of Medical Science, Kyoto Prefectural University of Medicine, 465 Kajii-cho, Kamigyo-ku, Kyoto 602-8566, Japan; ohgitani@koto.kpu-m.ac.jp (E.O.); masaharu@koto.kpu-m.ac.jp (M.S.-Y.); t-adachi@koto.kpu-m.ac.jp (T.A.); 4 Department of Dental Medicine, Graduate School of Medical Science, Kyoto Prefectural University of Medicine, Kamigyo-ku, Kyoto 602-8566, Japan; yamamoto@koto.kpu-m.ac.jp (T.Y.); kanamura@koto.kpu-m.ac.jp (N.K.); 5 Department of Orthopedic Surgery, Tokyo Medical University, 6-7-1 Nishi-Shinjuku, Shinjuku-ku, Tokyo 160-0023, Japan; 6 Department of Applied Science and Technology, Politecnico di Torino, Corso Duca degli Abruzzi 24, 10129 Torino, Italy; 7 Department of Molecular Science and Nanosystems, Ca’ Foscari University of Venice, Via Torino 155, 30172 Venice, Italy; 8 Department of Microbiology, School of Medicine, Kansai Medical University, 2-5-1 Shinmachi, Hirakata 573-1010, Japan; okumak@hirakata.kmu.ac.jp

**Keywords:** human herpesvirus, Herpes simplex virus, silicon nitride, surface hydrolysis, instantaneous inactivation, Raman spectroscopy, reverse transcription polymerase chain reaction

## Abstract

Hydrolytic reactions taking place at the surface of a silicon nitride (Si_3_N_4_) bioceramic were found to induce instantaneous inactivation of Human herpesvirus 1 (HHV-1, also known as Herpes simplex virus 1 or HSV-1). Si_3_N_4_ is a non-oxide ceramic compound with strong antibacterial and antiviral properties that has been proven safe for human cells. HSV-1 is a double-stranded DNA virus that infects a variety of host tissues through a lytic and latent cycle. Real-time reverse transcription (RT)-polymerase chain reaction (PCR) tests of HSV-1 DNA after instantaneous contact with Si_3_N_4_ showed that ammonia and its nitrogen radical byproducts, produced upon Si_3_N_4_ hydrolysis, directly reacted with viral proteins and fragmented the virus DNA, irreversibly damaging its structure. A comparison carried out upon testing HSV-1 against ZrO_2_ particles under identical experimental conditions showed a significantly weaker (but not null) antiviral effect, which was attributed to oxygen radical influence. The results of this study extend the effectiveness of Si_3_N_4_’s antiviral properties beyond their previously proven efficacy against a large variety of single-stranded enveloped and non-enveloped RNA viruses. Possible applications include the development of antiviral creams or gels and oral rinses to exploit an extremely efficient, localized, and instantaneous viral reduction by means of a safe and more effective alternative to conventional antiviral creams. Upon incorporating a minor fraction of micrometric Si_3_N_4_ particles into polymeric matrices, antiherpetic devices could be fabricated, which would effectively impede viral reactivation and enable high local effectiveness for extended periods of time.

## 1. Introduction

Herpes viruses are double-stranded DNA viruses that infect humans and other animals [1,2]. There are several types of herpes viruses, including Herpes simplex virus Type 1, Herpes simplex virus Type 2, Varicella-zoster virus, Epstein-Barr virus, Cytomegalovirus, and Herpesvirus 6, 7, and 8. Each has a slightly different molecular structure, but they share common metabolomic and infectivity features [2,3]. The general structure of herpes viruses consists of an icosahedral capsid that encloses the viral DNA genome. The capsid is composed of repeating subunits called capsomers, which are made up of viral proteins. A lipid bilayer envelope surrounds the capsid and embedded viral glycoproteins. These enveloped elements play crucial roles in viral entry into host cells [4], modification of the host cell membrane upon egress [5], and immune system evasion [6]. The herpesvirus genome is a linear double-stranded DNA molecule that is packaged inside the capsid; it contains multiple genes that encode various proteins involved in viral replication, assembly, and modulation of host immune responses.

The herpes virus, whose primary mode of transmission is through direct contact with infected bodily fluids or lesions, is highly resistant to environmental stresses and can survive for extended periods outside the body [7]. Its viral inactivation can be achieved through various strategies in support of natural host immune responses (by both innate and adaptive immune cells). These include antiviral medications and physical or chemical treatments [8,9]. A number of antiviral medications have been proposed for the treatment of Herpes virus infections [10,11,12,13]. Among these, medications referred to as acyclovir, valacyclovir, and famciclovir work by inhibiting the viral DNA replication process [14]. By targeting specific viral enzymes, these drugs prevent the virus from multiplying and spreading, ultimately leading to viral inactivation [15]. Physical treatments, such as ultraviolet light irradiation, can directly damage viral DNA, impairing the virus’s ability to replicate and infect host cells [16]. Various chemical agents have also been shown to inactivate the Herpes virus [17]. Common disinfectants, such as alcohol-based solutions, hydrogen peroxide, or quaternary ammonium compounds, can disrupt the viral envelope and inactivate the virus. Other chemicals, such as phenol or formaldehyde, can denature viral proteins or nucleic acids, leading to viral inactivation [18]. However, it is important to note that while these mechanisms can contribute to viral inactivation, complete eradication of the Herpes virus from the body is challenging. The virus can establish latency within nerve cells, evading immune responses and antiviral treatments. This latency allows the virus to reactivate periodically, causing recurrent outbreaks [19]. Moreover, to the authors’ knowledge, there has been no known method for instantaneously inactivating the herpes virus. Viral infection is thus a chronic condition in which the virus can remain in the body once a person is infected and goes through periods of activity (outbreaks) and periods of dormancy. Antiviral medications can help manage the symptoms and reduce the frequency and duration of outbreaks, but they do not eliminate the virus from the body.

In previous papers [20,21,22], micrometric silicon nitride (Si_3_N_4_) bioceramic particles were shown to be capable of instantaneously inactivating, upon contact, a host of different RNA viruses, including influenza strains, enteroviruses, caliciviruses, and several SARS-CoV-2 variants. Unlike ethanol (often used in combination with sodium hypochlorite), hydrogen peroxide, and ultraviolet light, the most popular virus-inactivating agents [12,17,23], the Si_3_N_4_ antiviral chemistry presents the unique advantage of being completely compatible with human cells. Accordingly, Si_3_N_4_ can continuously be used in contact with tissues and has been proposed as an alternative surface disinfectant against COVID-19 [20,21]. The discovery of the antiviral properties of Si_3_N_4_ [20,21,22] added important functionality to the application of this non-oxide ceramic as a biomaterial [24,25].

In this paper, we provide the first experimental proof for the potent antiviral efficiency of Si_3_N_4_ bioceramic particles against a DNA virus, namely, Herpes simplex virus Type 1 (HSV-1). This virus has long been studied in our laboratories [26,27]. To complement the phenomenological DNA fragmentation data collected using the standardized method of polymerase chain reaction, we systematically applied Raman spectroscopy to virions before and after Si_3_N_4_ exposure in an aqueous environment. While there has been extensive research on the molecular biology and genetics of herpes viruses, molecular-scale assessments are still relatively new and evolving. Following advanced deconvolutive procedures and a previously explained machine-learning approach [28,29,30], the Raman approach enabled us to clarify the antiviral mechanisms induced by instantaneous contact of herpes virions with ceramic particles at the molecular scale and in full detail. In summary, we show here that Si_3_N_4_ bioceramic particles of micrometric size, if incorporated into fabrics, adhesive bandage wraps, or antiviral creams and gels, could effectively support a therapy for extensive eradication of the Herpes virus through an instantaneous and continuative antiviral effect.

## 2. Results

### 2.1. Infectivity and Polymerase Chain Reaction Results

**Figure 1:**
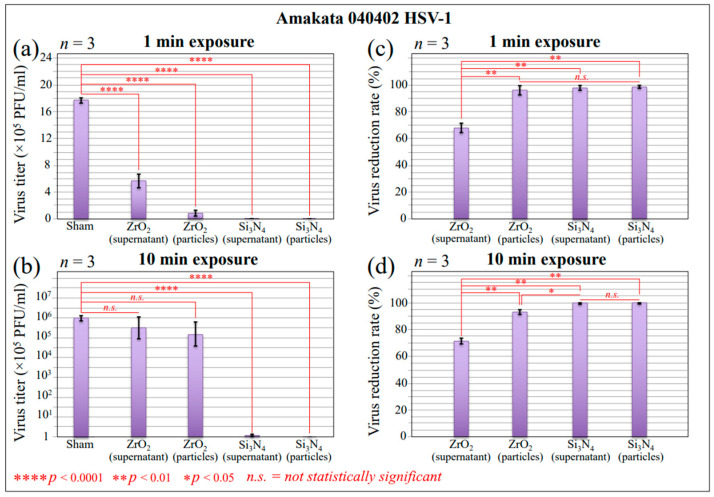
Results of immunochemical testing on Vero cells infected with HSV-1 virus after 1 min (**a**) or 10 min (**b**) exposure to Si_3_N_4_ or ZrO_2_ powders. All data are plotted (with statistical validation) in terms of PFU counts (cf. labels in the inset). Sham refers to a virus sample unexposed to ceramic particles. In (**c**) and (**d**), virus reduction percentage rates are plotted for virions exposed to ceramic particles for 1 and 10 min, respectively.

The effect of Si_3_N_4_ and ZrO_2_ surface contact on HSV-1 infection was analyzed using a Vero cell culture. Vero cells have been extensively used in virus research and cell-culture-based infection models because they support viral replication at high titers. This high susceptibility is related to the high expression level of the ACE-2 receptor [31] and its inability to produce interferon [32,33]. The virus was preliminarily exposed in aqueous solution to a fixed fraction of Si_3_N_4_ or ZrO_2_ powders (15 vol.%) and for two different exposure times (1 and 10 min; referred to as “virus inactivation times”, henceforth) prior to infecting the Vero cells. The particle fraction and the virion/particle contact times in suspension were selected based on previously optimized studies of the inactivation of Influenza and SARS-CoV-2 [20,21,22]. Si_3_N_4_ or ZrO_2_ particles were then removed through filtration and centrifugation, and the Vero cells were inoculated with virus samples for 48 h. Viral infectivity was compared to that of unexposed virions tested under exactly the same conditions as negative controls (sham samples). The plaque assay was applied as a standard method for determining virus concentration in terms of infectious dose. The number of plaques in cell samples was then plotted in terms of plaque-forming units (PFU/100 μL) for both inactivation times. Figure 1a,b shows the results (with statistical validation) of these experiments (*n* = 3) for HSV-1 virions exposed for 1 and 10 min to 15 vol.% Si_3_N_4_ or ZrO_2_ powders in solution, respectively. The corresponding reduction rates of infectious viruses for inoculation experiments conducted at room temperature are shown in Figure 1c and 1d, respectively. The results display data for both supernatant virions and virions on particles. PFU counts decreased dramatically for virions exposed to Si_3_N_4_ powder, with reduction rates comparable to the control. The reduction rate was as high as >98% (*p* < 0.001; *n* = 3) after only 1 min of exposure and >99% after 10 min of exposure. This experiment clearly demonstrated that the Si_3_N_4_ powder fully inactivated the HSV-1 virus within exposure times as short as 1 min. The effect of ZrO_2_ particles on virions tested under exactly the same conditions was significantly lower but not null, with a 68 and 71% virus reduction rate in the supernatant at 1 and 10 min exposures, respectively. The above results suggest the occurrence of chemical interactions at the virion/Si_3_N_4_ or ZrO_2_ interfaces in an aqueous environment. These reactions will be discussed in the forthcoming Section 3.1, Section 3.2 and Section 3.3. It is anticipated that inactivation mechanisms at various ceramic interfaces were intrinsically different and depended on the content of the respective hydrolytic reactions. Viral inactivation clearly affected the virions’ structure, as observed from the specific chemical reactions taking place instantaneously upon contact between the virus and ceramic particles.

**Figure 2:**
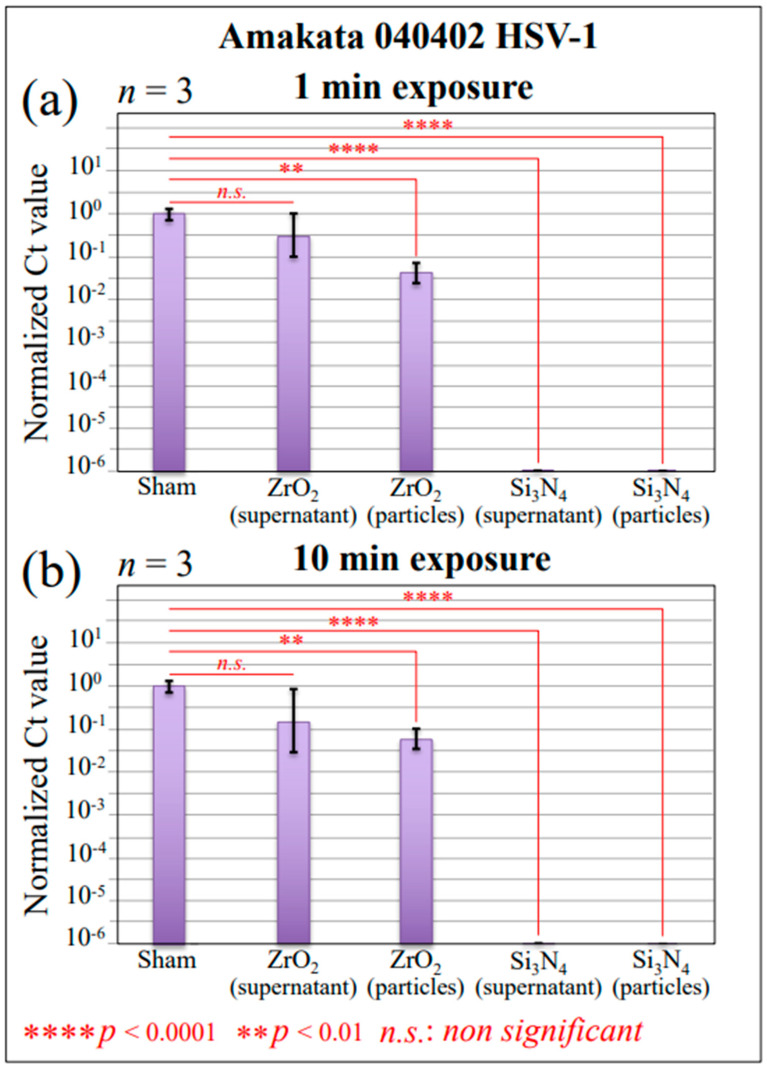
Results of RT-PCR tests to evaluate viral DNA fragmentation upon exposure to Si_3_N_4_ or ZrO_2_ ceramic powders for 1 and 10 min (in (**a**) and (**b**), respectively); a comparison of supernatants and powders in comparison with virions simply suspended in water is given using evaluations of viral DNA. Red inset labels in (**a**,**b**) comply with ANOVA variance statistics.

In order to measure the extent of viral DNA fragmentation upon contact with Si_3_N_4_ or ZrO_2_ powders, we conducted RT-PCR experiments on the virions’ gene sequence (Figure 2a,b) for 1 and 10 min exposures, respectively. Both supernatant virions and virions on pellets were tested in comparison with powder-unexposed control supernatant (a sham sample). As seen, both supernatant and particle virus DNA underwent severe damage, with a six-order-of-magnitude reduction in Ct value upon 1 min of Si_3_N_4_ contact. The same results were obtained after 10 min of exposure. On the other hand, 1 min of contact with ZrO_2_ particles induced insignificant fragmentation of supernatant virions and a two-order-of-magnitude Ct reduction on pellets (Figure 2a). This trend was not significantly improved upon increasing the exposure time to 10 min (Figure 2b). The combination of virus titer data and RT-PCR results unequivocally proves the occurrence of HSV-1 inactivation by Si_3_N_4_ bioceramic powder. Based on this evidence, we then attempted to clarify the inactivation mechanisms using Raman spectroscopy.

### 2.2. Raman Spectroscopic Results

**Figure 3:**
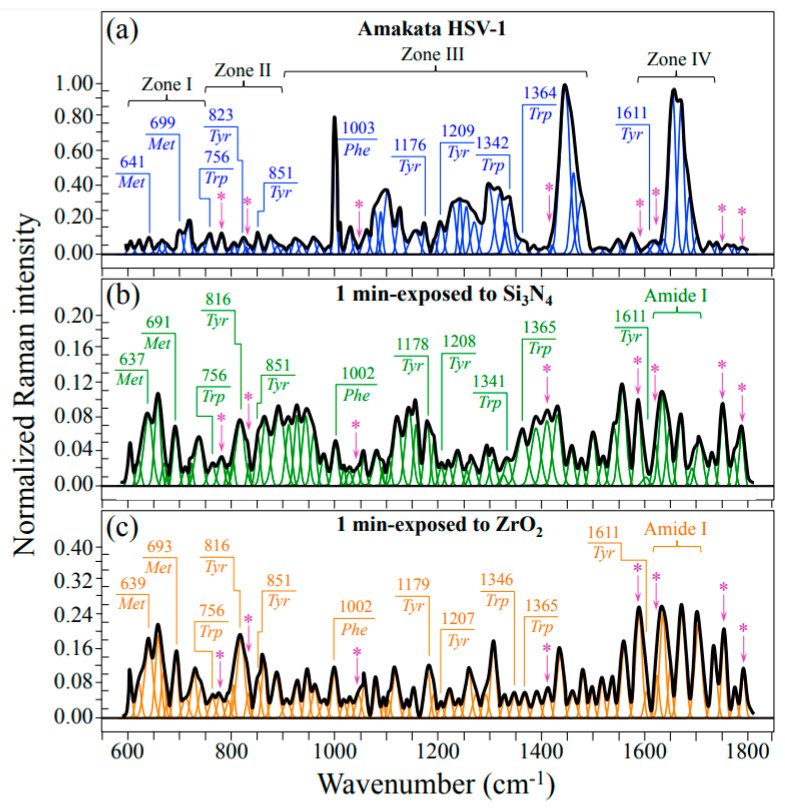
Raman spectra in the wavenumber interval 600~1800 cm^−1^ of (**a**) the pristine HSV-1 and of (**b**) and (**c**) of the same virus after exposure for 1 min in aqueous suspension to Si_3_N_4_ and ZrO_2_ particles, respectively. The spectra were normalized to their maximum signal and deconvoluted into Lorentzian-Gaussian sub-band components. Four zones are emphasized in (**a**), and labels show maximum wavenumbers for selected bands (the inset blue, green, and orange wavenumbers are in cm^−1^ units). Met, Tyr, Trp, and Phe are abbreviations for methionine, tyrosine, tryptophan, and phenylalanine, respectively. Additional vibrations related to structural modifications induced by exposure to ceramic powders are emphasized with pink asterisks, as mentioned in the text.

Figure 3 shows averaged and deconvoluted Raman spectra as collected in the wavenumber interval 600~1800 cm^−1^ on HSV-1 virions (a) before exposure (control sample), (b) and (c) after 1 min exposure in aqueous solution to 15 vol.% Si_3_N_4_ and ZrO_2_ powders, respectively. All spectra were normalized with respect to their strongest signals, namely, the CH_2_ vibrational bands at around 2940 cm^−1^ (not shown in Figure 3). The spectra collected before and after exposure appeared very different for both ceramic powders, suggesting the occurrence of fundamental modifications in the virion structure at the molecular scale. Spectral differences were discussed with respect to four selected spectral zones labeled as Zones I~1V and located at 600~750 cm^−1^, 750~900 cm^−1^, 900~1450 cm^−1^, and 1600~1750 cm^−1^. Figure 3’s red and blue inset labels indicate selected spectral zones and signal wavenumbers from the main amino acid residues present in the virion structure. Raman spectra in selected spectral zones were then collected with higher spectral resolution, normalized, and deconvoluted into sub-band components according to the computational procedure discussed in the forthcoming Section 3.4.

**Figure 4:**
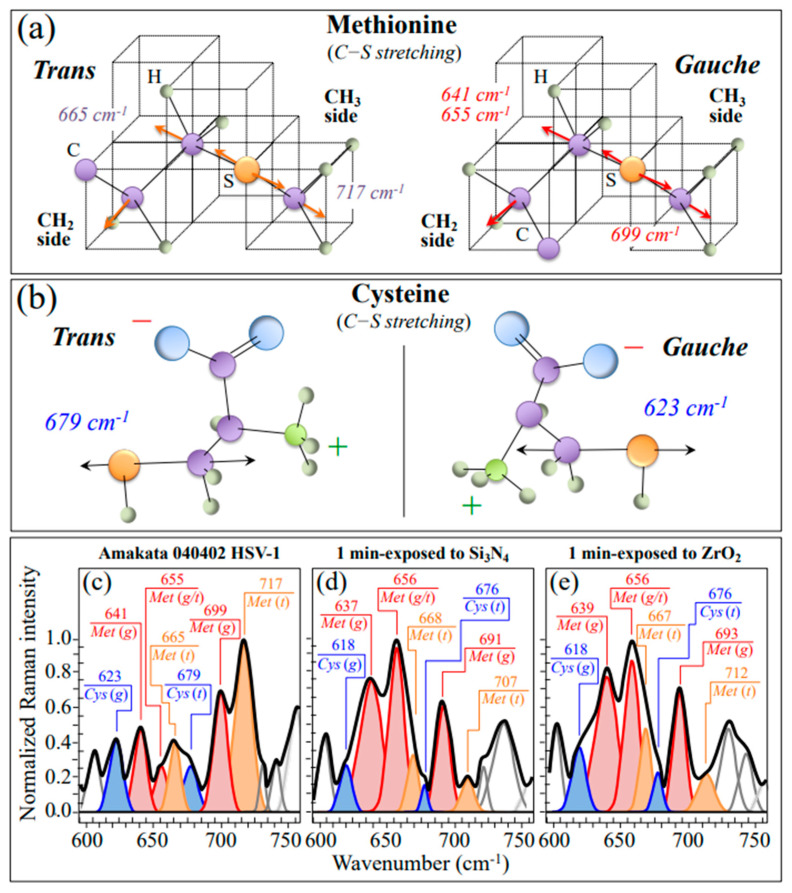
Schematic drafts of (**a**) methionine (*Met*) and (**b**) cysteine (*Cys*) *trans* and *gauche* rotamers with related vibrational stretching modes. In (**c**), (**d**), and (**e**), highly resolved Zone I of the HSV-1 spectrum before and after 1 min exposure in aqueous suspensions of Si_3_N_4_ and ZrO_2_ micrometric powders, respectively; spectra are deconvoluted into a sequence of Lorentzian-Gaussian sub-band components (the inset wavenumbers are in cm^−1^ units).

Figure 4a,b shows schematic drafts of different rotamers of methionine (Met) and cysteine (Cys) residues, respectively, with their respective C−S stretching vibrational modes and related wavenumbers. Met and Cys rotamers (t and g for trans and gauche, respectively) are the only sulfur-containing amino acids present in viral proteins. The spectra in sections (c), (d), and (e), which refer to the spectroscopic Zone I, are from virions before exposure (control sample) and after 1 min exposure to Si_3_N_4_ and ZrO_2_ powders, respectively (cf. also labels). Zone I is dominated by vibrational signals related to the C−S stretching bonds [34,35,36], whose relative intensities can be taken as representative of the volume fractions of the respective rotamers. The significant morphological differences between the control virus sample (Figure 4c) and virus samples exposed for 1 min to ceramic powders prove the occurrence of profound structural changes in Met residues. On the other hand, a much lesser variation was noticed in Cys-related bands. Note that Raman spectroscopy is extremely sensitive to structural modifications of sulfur-containing amino acid residues because of its high sensitivity to C−S bond vibrations. C−S stretching vibrations on the CH_3_ side of the Met molecule give rise to two Raman bands at ~717 and ~699 cm^−1^, which could be attributed to t and g rotamers, respectively [37]. On the other hand, the C−S signal scattered from the CH_2_ side of the molecule is found at ~655 cm^−1^ and belongs to both rotamers, while the C−S + S−C in-phase stretching mode appears at the distinct frequencies of 669 and 641 cm^−1^ in t and g molecular configurations, respectively. Exposure to either Si_3_N_4_ or ZrO_2_ surface environments stressed the virus structure and induced dramatic changes in the population ratio of the two Met rotameric forms. Common features were noticed for exposure to different ceramic powders: (i) a strong increase in signal intensities at 655 and 641 cm^−1^ (with the latter shifted to lower wavenumbers), and (ii) a significant intensity decrease and shift to lower wavenumbers for the signal at 717 cm^−1^. The concurrent increase in relative intensity of the two shoulder bands at 1043 and 1623 cm^−1^ as compared to the spectrum of unexposed virions (cf. arrowed asterisks in Figure 3c), which can be assigned to S=O stretching and NH_3_^+^ in-plane bending, respectively, points to the formation of methionine sulfoxide. This agrees with a significant enhancement of two bands from –COO^−^ terminal bonds, whose symmetric and antisymmetric stretching vibrations display at 1414 and 1590 cm^−1^, respectively (cf. asterisks in Figure 3). Note that the latter band overlaps the in-plane bending vibrations of the NH_3_^+^ terminal group in methionine sulfoxide [38]. As discussed in more detail in Section 4, such spectroscopic variations point to a dramatic post-translational modification of proteins as engendered by the presence of non-radical and free radical species [39,40].

**Figure 5:**
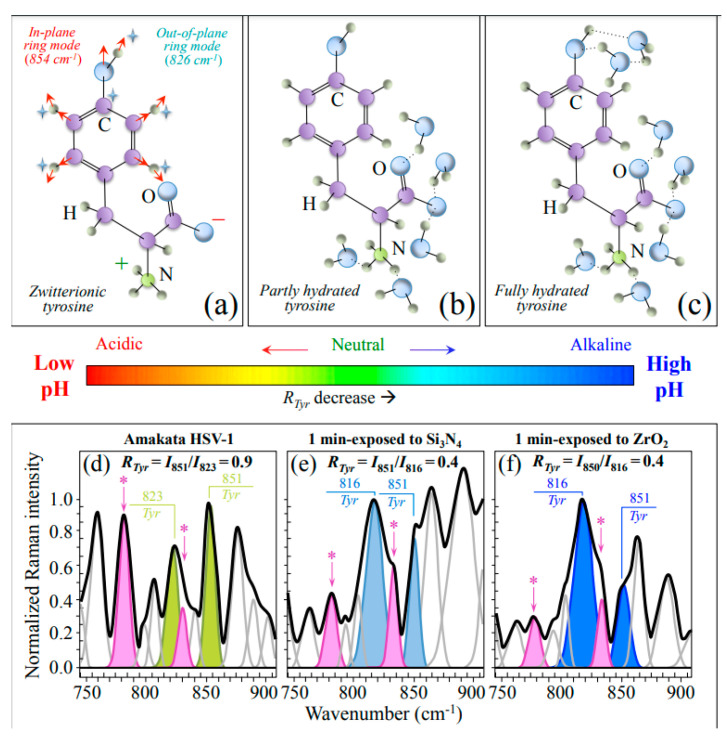
Schematic drafts of (**a**) zwitterionic, (**b**) partly hydrated, and (**c**) fully hydrated tyrosine (*Tyr*) molecules. In (**d**), (**e**), and (**f**), a highly resolved Zone II of the HSV-1 spectrum indicates the virus before and after 1 min exposure in aqueous suspension to Si_3_N_4_ and ZrO_2_ micrometric powder, respectively; spectra are deconvoluted into a sequence of Lorentzian-Gaussian sub-band components (the inset wavenumbers are in cm^−1^ units; values of Raman ratios *R_Tyr_* = *I*_851_/*I*_823_ are given inset). Additional vibrations related to structural modification upon exposure to ceramic powders are emphasized with asterisks as discussed in the text.

Zone II (Figure 5) contains a doublet from tyrosine (also referred to as a “Fermi doublet”), which displays at 851 and 823 cm^−1^. The doublet intensity ratio, referred to as *R_Tyr_* = I_851_/I_823_, is diagnostic of the H-bonding environment around the tyrosine units; the lower the ratio, the higher the pH, and the more hydrophobic the environment in which the tyrosine residue is embedded [41,42]. Figure 5a shows the vibrational origins of the tyrosine doublet as arising from two independent modes of the phenol ring: in-plane ring breathing and out-of-plane C−H bending (at 851 cm^−1^ and 823 cm^−1^, respectively). In sections (b) and (c) of the same figure, partly hydrated and fully hydrated tyrosine molecules are shown, respectively. As it is a sensor of hydrophobic/hydrophilic balance in environmental interactions, such altered configurations of the tyrosine molecule greatly affect the *R_Tyr_* intensity ratio. The spectroscopic response of the tyrosine doublet before and after exposure to Si_3_N_4_ and ZrO_2_ powders is shown in Figure 5d, 5e, and 5f, respectively. A clear trend inversion for the doublet could be observed upon virus exposure to both ceramic powders, with the intensity ratio, *R_Tyr_*, changing from 0.9 (for the virus control sample) to 0.4 (for the virus on both ceramic powders). This bold variation arises from a perturbation of the benzene ring symmetry, which causes an intensity increase and shift of the low-frequency band towards lower wavenumbers (i.e., from 823 to 816 cm^−1^; cf. Figure 5e,f), as a fingerprint of pH alkalinization of the environment at the interface between the virion and its environment.

**Figure 6:**
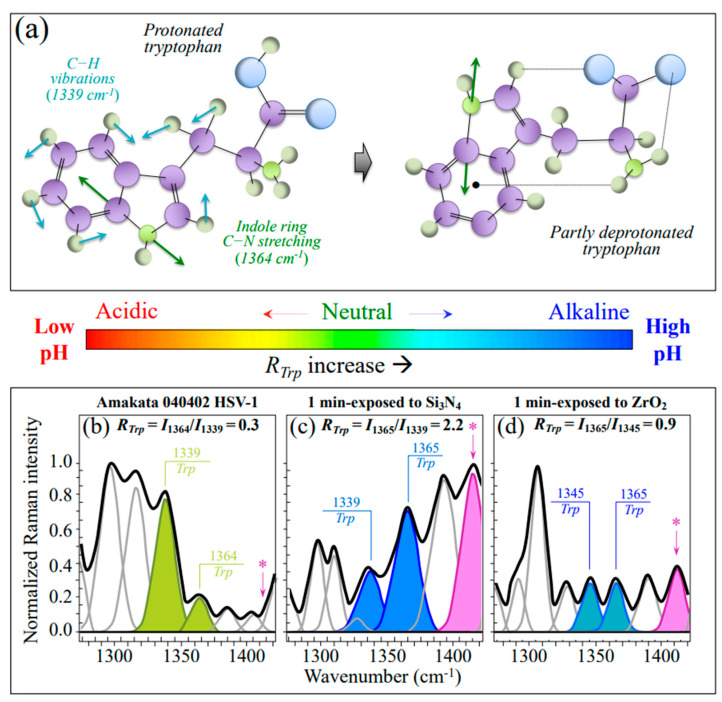
Schematic drafts of (**a**) protonated and partly deprotonated tryptophan (*Trp*) molecules. In (**b**), (**c**), and (**d**), highly resolved spectra of the HSV-1 virus (in the wavenumber interval 1280~1420 cm^−1^) before and after 1 min exposure in aqueous suspension to Si_3_N_4_ and ZrO_2_ micrometric powders, respectively, are deconvoluted into a sequence of Lorentzian-Gaussian sub-band components (the inset wavenumbers shown in green, blue, and royal blue are in cm^−1^ units; inset values are of Raman ratios *R_Trp_* = *I*_1364_/*I*_1339_). Additional vibrations related to structural modification upon exposure to ceramic powders are emphasized with asterisks as discussed in the text.

Analyses of the tryptophan doublet before and after exposure to ceramic powders gave an additional spectroscopic signature for the increased virion-solid interface pH. The tryptophan doublet consists of signals at 1340 and 1361 cm^−1^, which arise from C−H bending and indole ring stretching, respectively [43]. Similar to the *R_Tyr_* doublet ratio, the tryptophan doublet ratio, *R_Trp_* = I_1361_/I_1340_, is also an indicator of hydrophobicity and thus links to environmental pH [43,44]. The 1361 and 1340 cm^−1^ bands are stronger in both hydrophobic and hydrophilic environments. The higher the *R_Trp_* value, the higher the pH, and the more hydrophobic the environment in which the tryptophan residue is embedded. Figure 6a shows a draft of the tryptophan structure and its (partly) deprotonated structure, together with the tryptophan doublet vibrations, as described above. In Figure 6b–d, a tryptophan doublet is apparent for the pristine HSV-1 virus sample as well as for virus samples exposed to Si_3_N_4_ and ZrO_2_ powders. The tryptophan doublet ratio, *R_Trp_*, experienced more than sevenfold (2.2 vs. 0.3) and threefold (0.9 vs. 0.3) increases after 1 min of exposure to Si_3_N_4_ and ZrO_2_ powders, respectively, again proving the alkaline shifts of viruses exposed to ceramic powders.

**Figure 7:**
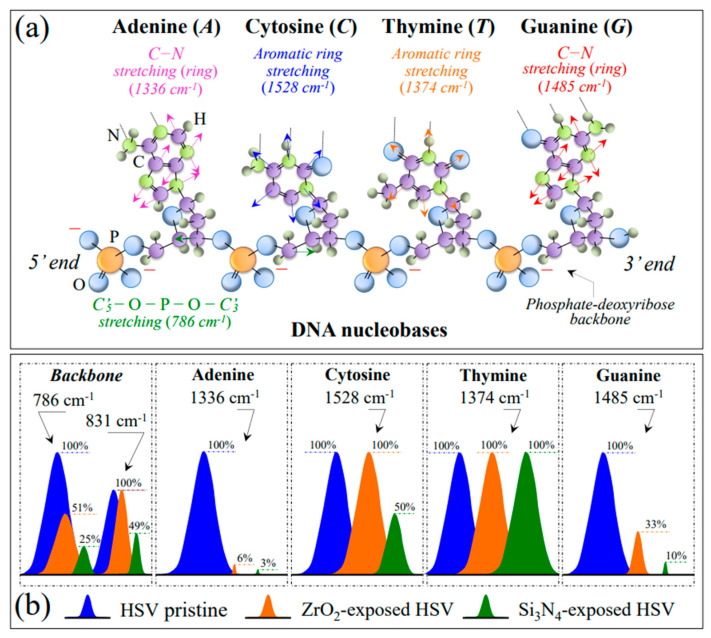
(**a**) Schematic draft of linked DNA nucleobases with wavenumbers of their respective ring vibrational fingerprints (cf. labels); in (**b**), comparison between relative intensities of fingerprint signals for phosphate-deoxyribose backbone, adenine, cytosine, thymine, and guanine (cf. labels in (**a**)) in the spectrum of unexposed HSV-1 virions (taken as 100%) and spectra of HSV-1 virions after 1 min exposure in aqueous suspension to Si_3_N_4_ and ZrO_2_ micrometric powders (cf. intensity reductions indicated in colored inset labels).

**Figure 8:**
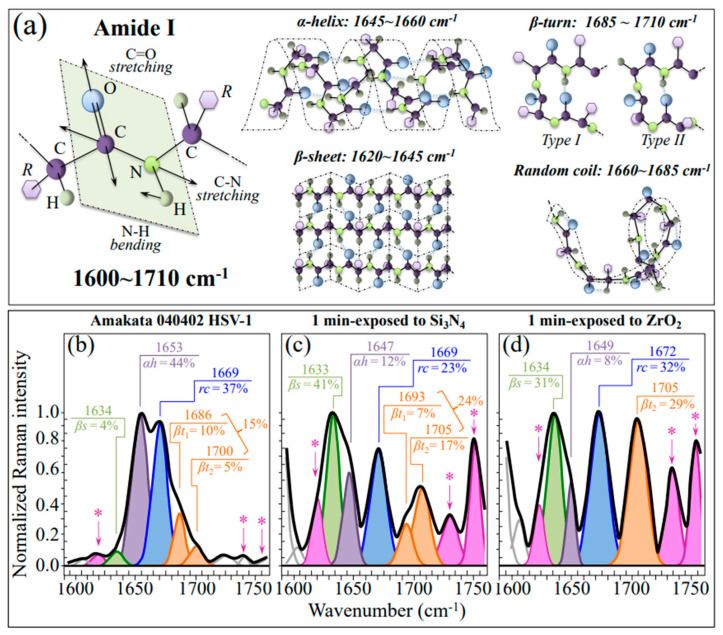
(**a**) Schematic drafts of Amide I vibrations and secondary structures of proteins with their relative wavenumber intervals. In (**b**), (**c**), and (**d**), highly resolved spectra of the HSV-1 virus in Zone IV (1600~1750 cm^−1^) before and after 1 min exposure to Si_3_N_4_ and ZrO_2_ micrometric powders in aqueous suspension, respectively (cf. labels in inset); spectra are deconvoluted into a sequence of Lorentzian-Gaussian sub-band components (the inset wavenumbers are in cm^−1^ units; abbreviations βs, αh, rc, βt_1_, and βt_2_ refer to β-sheet, α-helix, random coil, and *Type I* and *Type II* β-turn rotamers, respectively). Inset labels also give the relative fractions of different secondary structures. Additional vibrations related to structural modification upon exposure to ceramic powders are emphasized with asterisks as discussed in the text.

Zone III contains a prominent Raman signal from phenylalanine (Phe) at 1004 cm^−1^ (symmetric ring breathing) and incorporates ring-related signals mainly assignable to DNA purines and pyrimidines (cf. Figure 3). Bands representing stretching of heterocyclic aromatic rings that belong to cytosine (C) and thymine (T) pyrimidines could be found at 1528 and 1374 cm^−1^, respectively [45,46,47]. On the other hand, adenine (A) and guanine (G) purines display cumulative C–N stretching in the imidazole and pyridine rings at 1336 and 1485 cm^−1^, respectively [48,49]. Additional features related to the DNA structure are the signals at ~786 and ~831 cm^−1^, which both appear in Zone II (cf. asterisked bands in Figure 3 and Figure 5d). These latter signals are mainly contributed by symmetric and antisymmetric stretching modes of C–O–P–O–C phosphodiester bonds in the phosphate-deoxyribose backbone [50]. Figure 7a gives a draft of the linked structure of DNA nucleobases with the related Raman spectroscopic fingerprints. As shown in Figure 7b, a feature commonly found when HSV-1 virions were exposed for 1 min either to Si_3_N_4_ or ZrO_2_ powders was a significant reduction in the relative Raman intensities of both purine nucleobases; in contrast, a much lower, or even null, relative intensity reduction could be observed for signals representing pyrimidines. Regarding the trend observed for C–O–P–O–C phosphodiester bonds in Si_3_N_4_ and ZrO_2_ exposed virions, the Raman data agree with the extent of fragmentation in viral DNA recorded by RT-PCR analyses on supernatant virions (cf. Figure 2a and Figure 7b backbone). The strong reductions of A and G ring signals upon short-term exposure to the ceramic powders hint at the occurrence of DNA damage in the form of ring disruption. However, breakage of the imidazole ring to form formamidopyrimidines cannot directly occur through interaction with ammonia molecules; rather, it can only be induced by off-stoichiometric reactions with oxygen or nitrogen radicals [51]. This observation agrees with the post-translational modifications in the methionine residues (as observed in spectral Zone I), which, to occur, also necessitate the action of free radicals (cf. above). The significant reduction observed in the phenylalanine ring vibration at ~1003 cm^−1^ (cf. Figure 3a–c) likely has the same radical-related molecular origin. Douki and Cadet [52] showed that peroxynitrite anions (O=NOO^−^) can exhibit pronounced oxidizing action toward purine moieties, which in turn leads to very low 8-oxo-7,8–dihydro–2′–deoxyguanosine (8–oxo–dGuo) production. In a subsequent work, Douki et al. [53] linked the presence of 8–oxo–dGuo to the formation of 2,6–diamino–4–hydroxy–5–formamidopyrimidine and extended the findings to the oxidation product of the adenine base, 4,6–diamino–5–formamidopyrimidine. The significant enhancement in C=O stretching vibrations at >1730 cm^−1^ (cf. bands labeled with asterisks in Figure 3 and Figure 8b–d) could be seen as proof of oxidized purine molecule formation. The hypothesis of radical formation at the virion/ceramic interface will be discussed in more detail in Section 4. On the other hand, in addition to ring disruption in purines, the present Raman study also revealed lesser or null variations in the cytosine and thymine signals. As they are quite insensitive to pH variations [54], degradation of the virtually stable cytosine and thymine ring structures can only arise from photohydration reactions [55], which in turn require high-energy radiative, oxygen-independent processes [56]. Such processes are clearly absent from the hydrolytic reactions in question here. Moreover, Nonoyama et al. [57] have reported that cytosine is almost inert in response to the peroxynitrite anions. This indirectly supports our present interpretation of the presence of nitrogen radicals. The above references [54,55,56,57] provide the basis for interpreting the present Raman findings of purine molecule oxidation in contraposition to the high/full inertness of pyrimidines embedded in a ceramic hydrolytic environment.

Finally, Zone IV covers the Amide I wavenumber region, which is representative of the secondary structures of viral proteins (Figure 8a). The deconvoluted Amide I band components from proteins in the control HSV-1 sample are assigned to β-sheet (βs; at ~1634 cm^−1^), α-helix (αh; at ~1653 cm^−1^), random coil (rc; at ~1699 cm^−1^), and Type I and Type II β-turn rotamers (βt_1_ and βt_2_; at ~1686 and ~1700 cm^−1^, respectively) (Figure 8b) [58]. Comparing pristine (Figure 8b) and Si_3_N_4_- or ZrO_2_-exposed virions (Figure 8c and d, respectively) revealed a marked common variation: a quite strong relative increase in β-sheet and β-turn fractions (cf. relative fractions in inset). However, while in Si_3_N_4_-exposed virions the fractional increase in β-sheet and β-turn occurred at the expense of both α-helix and random coil, in ZrO_2_-exposed virions only a significant reduction in α-helix was recorded, while the fraction of random coiling increased to the same level as that of β-sheet. The β-turn rotamers were also significantly reshuffled upon contact with the ceramic powders, with an inversion in the rotamers’ fractional trend for Si_3_N_4_-exposed virions and a reduction to only Type II in the ZrO_2_-exposed ones. Raman spectroscopy represents a unique tool in assessing post-translational modifications in proteins and is widely exploited in therapeutic production because fine structural rearrangements are virtually undetectable using conventional protein analysis [59]. Post-translational modifications of viral proteins have recently been reviewed by Fung and Liu [60], with emphasis on their impact on viral replication and pathogenesis. Unfortunately, Raman spectroscopic assessments can hardly link the observed changes in secondary protein structures to the specific envelopment proteins responsible for the observed loss of viral infectivity. However, rotameric variations observed in S-containing amino acids could help in interpreting variations in secondary protein structures since disulfide bond formation likely governs folding and trafficking of envelope proteins. The observed rotameric modifications in methionine rotamers (Zone I; cf. Figure 4) should be related to the unfolding of the α-helical structure after hydrolytic reaction interaction at the solid interface because of the high helix-forming propensity of methionine [61]. A recent computational simulation study of the impact of methionine oxidation on the dynamics and energetics of protein α-fold conformation has located helical methionine sulfoxidation as a strong trigger in destabilizing the native α-fold configuration while leading to protein conformational switch into β-sheet configuration [62]. This change provides a more flexible secondary structure, but it also favors alternative states that might be essential in the observed viral inactivation pathway.

## 3. Discussion

### 3.1. Hydrolytic Reactions and Kinetics of Si_3_N_4_ in an Aqueous Environment

Si_3_N_4_ undergoes hydrolysis and minute (but continuous) surface dissolution when exposed to water vapor or when embedded in an aqueous environment. The hydrolytic reaction, which ultimately leads to the formation of a surface oxide layer, releases ammonia according to the following chemical reaction:

Si_3_N_4_ + 6H_2_O → 3SiO_2_ + 4NH_3_ (1)

Although variability can be encountered among commercially available Si_3_N_4_ powders or even between individual batches due to differences in stoichiometry and in the extent of surface oxidation, the homolytic dissociation of Si–N covalent bonds in an aqueous environment is an unavoidable effect. This reaction liberates nitrogen anions, which strongly attract H^+^ cations, leading to local pH buffering at the solid/liquid interface [63]. A direct consequence of nitrogen elution is the increased electrophilicity at the adjacent silicon sites, which leads to nucleophilic attack by water attempting to form silanol complexes. However, silanols are metastable and promptly decay with the liberation of Si ions at an environmental-pH-dependent ratio; the higher the pH, the higher the silanol solubility [64]. The formation of ammonia (NH_3_) takes place in tandem with the formation of silicon dioxide (SiO_2_) on the solid surface. Successive hydrolysis progression produces ammonium (NH_4_^+^) and orthosilicic acid (Si(OH)_4_), according to the two following reactions:

H^+^ + NH_3_ → NH_4_^+^ (2)

SiO_2_ + 2H_2_O → Si(OH)_4_ (3)

The formation of NH_4_^+^ from NH_3_ (i.e., according to Equation (2)) strongly depends on pH. At room temperature, the fraction of NH_3_ varies with pH according to a sigmoidal dependence, as follows [65,66]:

[NH_3_][H^+^]/[NH_4_^+^] = 5.7 × 10^−10^ (4)

This relationship indicates that the relative fraction of free NH_3_ in solution at physiological pH is quite low (i.e., 1~2% of the overall amount of ammonium species eluted). However, it increases linearly up to pH~9.3 and then steeply rises to ~100% NH_3_ at pH > 10. Relative concentrations of NH_3_ and NH_4_^+^ as a function of pH were experimentally measured for pure water containing dilute fractions of Si_3_N_4_ powder according to colorimetric ammonia assays [63]. This set of independent data validated Equation (4) and showed that the addition of ≥15 vol.% Si_3_N_4_ powder led to a stable pH value ~8.3, corresponding to ~7% NH_3_ (i.e., quantitative concentrations of NH_3_ and NH_4_^+^ equal to 0.06 and 0.35 μMol/dm^3^, respectively).

An important characteristic linked to the kinetics expressed by Equation (4) is the dualistic effect of Si_3_N_4_ surface chemistry on eukaryotic cells and pathogens; it is supportive of the former and harmful to the latter [63]. Ammonium, NH_4_^+^, can enter the cytoplasmic space of eukaryotic cells only through specific channels, and, being physiologically modulated by intracellular pH, it is used by cells in their nutritional cycle. Conversely, ammonia (NH_3_) is a highly volatile molecule that freely penetrates the cell membrane. It requires oxygen radicals to be oxidized into hydroxylamine, NH_2_OH, through a reaction referred to as enzymatic ammonia monooxygenase catalysis. Unpaired electrons, which can react with adsorbed O_2_ to yield •O_2_^−^ radical anions and other highly reactive oxygen species (ROS), are preponderantly contributed by cellular mitochondria. The mitochondrion produces oxygen radicals as “by-products” of partially reduced species upon leaking electrons from its transport chains, according to cytochrome c oxidase enzymatic catalysis [67]. Leaked free electrons can thus oxidize small concentrations of exogenous NH_3_ into NH_2_OH with successive occurrences of hydroxylamine oxidoreductase, leading to the formation of nitric oxide NO radicals, a key radical in eukaryotic cell signaling and multiplication [68]. Note that the threshold NO concentration leading to cell apoptosis is about three orders of magnitude higher than that induced upon local elution of ammonia from Si_3_N_4_ [69]. This can explain why the dissolution kinetics of Si_3_N_4_ are friendly to the human body, as demonstrated in previous studies [63,70,71,72].

Although different cells might differ in their ability to exploit ammonia as an alternative nitrogen source [73], eukaryotic cells generally proliferate in the presence of Si_3_N_4_ because mitochondria help maintain equilibrium between eluted and metabolized nitrogen. Conversely, viruses and prokaryotic cells lack mitochondria, and therefore the cocktail of eluted NH_4_^+^ ions and NH_3_ molecules becomes lethal to them due to the creation of an unmetabolized surplus of nitrogen moieties in the intracellular or intraviral space. The direct interaction of ammonia with other structural macromolecules produces a cascading reaction ending with the formation of toxic concentrations of highly reactive nitrogen radicals, which include nitric oxide and peroxynitrite (O=N–O–O^−^) moieties [74]. These reactive nitrogen species (RNS) irreversibly degrade DNA, with the degradation mechanisms mainly consisting of a strong oxidation effect that concurrently leads to the opening of the guanine ring, significant structural modifications of the adenine ring, and cleavage of phosphodiester DNA backbone linkages [74], which is precisely what we observed in the present experiments. DNA degradation mechanisms induced in HSV-1 virions by instantaneous contact with Si_3_N_4_ powder are discussed in detail in the next section according to Raman spectroscopic assessments of Si_3_N_4_-exposed virions.

Another important aspect of Si_3_N_4_ surface chemistry in virus interaction resides in the metastability of surface silanols. According to molecular dynamics simulations [64], an unstable intermediate molecular complex involving a penta-coordinated silicon forms upon hydrolysis on the Si_3_N_4_ surface, according to the following reaction:

Si(OH)_4_ + (OH)^−^ → [Si(OH)_5_]^−^ → [(OH)_3_SiO]^−^ •H_2_O (5)

The activation energy for the above reaction is also pH dependent and significantly decreases when surface silanols at Si–O–Si bridges switch from a protonated (Si–OH_2_^+^) state to a neutral (Si–OH) state and then to deprotonated (Si–O^−^) ones with increasing environmental pH. In other words, a shift toward alkaline pH values facilitates the elution of silanols by enhancing their solubility in an aqueous environment. In biological environments, the possibility of periodically refreshing the surface by restarting ammonia and silicic acid elution is a uniquely important characteristic. It ensures the long-term effectiveness of bioactive reactions once the material contacts tissue or microorganisms. An additional benefit arises from the fact that micrometric Si_3_N_4_ particles are fully resorbable in the biological environment and will dissolve within a month without contaminating human tissues for long periods of time [75,76].

### 3.2. Mechanisms of Viral Inactivation by Si_3_N_4_ Ceramic Powder

Following the discussion of the previous section, the main consequences of hydrolysis at the Si_3_N_4_ surface could be summarized as follows: (i) homolytic dissociation of Si–N covalent bonds with elution of N and successive formation of NH_3_/NH_4_^+^ species (in relative fractions depending on environmental pH); (ii) subsequent shift of environmental pH toward stable alkaline values ~8.3; and (iii) formation of deprotonated silanols at the solid surface (increasingly dissolving in aqueous solution as pH increases). On the virions’ side, the above chemical features are expected to: (i) alter the pH at the virion surface; (ii) increase the production of ROS and RNS; damage the DNA structure; and (iii) induce protein denaturation while altering their secondary structure [76,77,78,79]. This section discusses in detail the vibrational fingerprints of structural degradation as observed in HSV-1 virions upon Si_3_N_4_ exposure.

#### 3.2.1. Vibrational Proofs of Enhanced pH at the Virion Surface

One main spectral feature proving the occurrence of an alkaline environmental shift at the HSV-1 virion surface (as compared to the control) is represented by the spectroscopic behavior of the characteristic tyrosine doublet ratio, *R_Tyr_* = I_851_/I_823_. The tyrosine (or Fermi) doublet at 823 and 851 cm^−1^ arises from out-of-plane C–H bending and in-plane ring breathing vibrations, respectively. Quite sensitive to the surrounding environment, it provides information on the chemical environment in the immediate neighborhood of the virions [80]. More specifically, the intensity ratio, *R_Tyr_*, is diagnostic of the H-bonding environment around tyrosine residues; the lower the ratio, the more hydrophobic the tyrosine configuration. In other words, an increasingly alkaline environment increases tyrosine hydrophobicity and vice versa for an acidic environment; accordingly, the lower the *R_Tyr_* value, the higher the extent of surface protonation, the more alkaline the environment, and the more hydrophobic the state of proteins. As shown in Figure 4, HSV-1 treated with Si_3_N_4_ powder showed a more than twofold decrease in tyrosine ratio as compared to control virions (*R_Tyr_* = 0.4 vs. 0.9). Accordingly, the Raman response of tyrosine residues proved a significant pH shift of the virions’ surface towards alkalinization.

An important confirmation of the alkalinization of the virion surface could be obtained upon monitoring the relative intensity of the tryptophan doublet at 1340 and 1361 cm^−1^ (*R_Trp_* = I_1361_/I_1340_), which is another indicator for hydrophobicity and thus links to environmental pH [43,44]. In this additional spectroscopic pH fingerprint, signals at 1340 and 1361 cm^−1^ arise from C–H bending and indole ring stretching, respectively [43]. As previously mentioned, the 1361 and 1340 cm^−1^ bands are stronger in hydrophobic and hydrophilic environments, respectively. Figure 5a shows drafts of protonated and partly deprotonated tryptophan structures with vibrational modes related to the *R_Trp_* doublet. The HSV-1 virions exposed to Si_3_N_4_ experienced a nearly one order of magnitude higher tryptophan ratio as compared to control virions (*R_Trp_* = 2.2 vs. 0.3; cf. Figure 5b,c) and labels in inset), thus confirming the strong alkaline pH shift recorded by *R_Tyr_* monitoring in Si_3_N_4_-exposed virions.

Lancz [81] studied the relationships between the inactivation rates of Herpes simplex virus types 1 and 2 and the pH and composition of the suspending medium. Clear decrements in viable virions were recorded when the medium pH was adjusted to 6.3 or 7.8. The maximum decrease (i.e., ~4.5 log 10) could be observed at the highest pH after incubation at 36° for 48 h in tissue culture medium. However, the stability of HSV virions suspended at pH 7.0 was somewhat enhanced. The present results are in line with Lancz’s findings by indicating that full inactivation of HSV-1 could occur at pH~8.3, as established at the contact interface with the Si_3_N_4_ solid surface.

#### 3.2.2. Oxidation of S-Containing Amino Acid Residues

As discussed in our previous Raman study of virus structures [21], Raman spectroscopy is uniquely positioned to capture such structural modifications in S-containing amino acids because of its high sensitivity to C–S bond vibrations. As shown in the drafts in Figure 4a, different signals in the low-frequency region arise from C–S stretching modes in different methionine rotamers, namely, the trans and gauche rotamers. Similar differences are observed in the trans and gauche rotamers of cysteine (cf. Figure 4b) [37,82]. In the Raman spectrum of the pristine HSV-1 virions (Figure 6c), the most intense methionine band was found at ~717 cm^−1^, suggesting that methionine residues are mainly in the trans-rotameric configuration. Exposure to the Si_3_N_4_-solid interface stressed the protein structure and induced a change in the population ratio with a preponderance of the gauche rotamer (cf. change from 1.7 to 0.3 of the intensity ratios between the 717 cm^−1^ band, shifted to 707 cm^−1^, and the 699 cm^−1^ band, shifted to 691 cm^−1^). The concurrent intensity reduction of the trans signal at 665 cm^−1^ confirms this trend.

**Figure 9:**
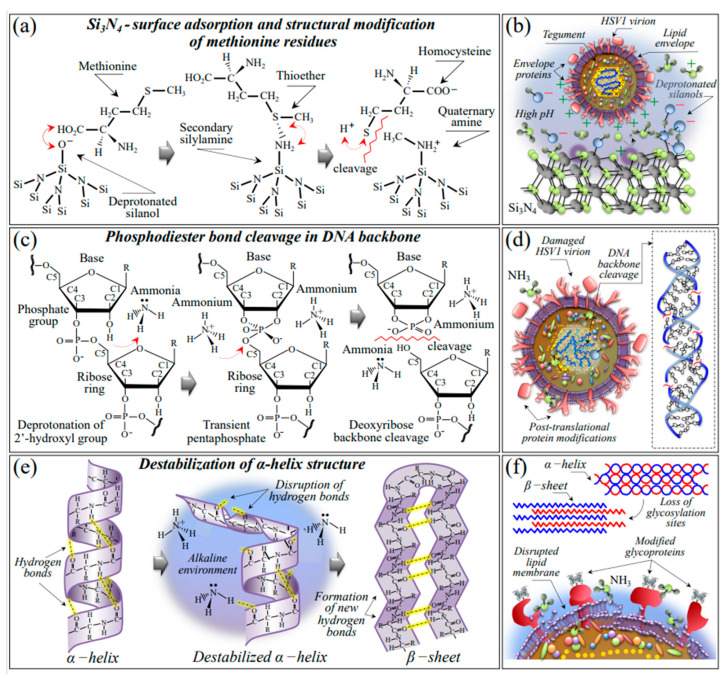
Schematic drafts of: (**a**) the proposed interaction between methionine residues contained in the envelope glycoproteins of HSV-1 virions and deprotonated silanols formed in the highly alkaline environment at the Si_3_N_4_ solid surface, and (**b**) the interaction between HSV-1 solid surface interaction upon Si_3_N_4_ hydrolysis. The most probable scenario behind the electrostatic attraction between Si_3_N_4_ surface and envelope proteins involves deprotonated silanol groups at the surface of Si_3_N_4_ strongly attracting the C-COOH terminus of methionine and cysteine residues. A schematic diagram of the chemical reactions leading to DNA backbone cleavage (**c**) and a draft of damaged HSV-1 undergoing a structural order → disorder transition due to the presence of ammonia (**d**); backbone damage starts with deprotonation at the 2′-hydroxyl group by NH_3_, proceeds with destabilization of the ribose ring chain with the formation of a transient pentaphosphate, and ends with bond cleavage upon interaction with acidic NH_4_^+^ ions. (**e**) The destabilization process of the *α*-helix structure as triggered by the disruption of its hydrogen bonds in a highly alkaline environment; and (**f**) the draft of the protein structural modifications from α-helix to β-sheet, ultimately leading to the loss of glycosylation sites in envelope glycoproteins.

Although destabilization of the protein structure (as discussed later in more detail) is obviously expected to scramble rotameric symmetry, additional fingerprint features found in the methionine spectrum of Si_3_N_4_-treated virions point to oxidation of both sulfur amino acid residues. Exposure of HSV-1 virions to the Si_3_N_4_ solid surface induced a significant increase in the relative intensity of the methionine (gauche) doublet at ~640 and ~655 cm^−1^, including an upturn of their relative intensity. The band at ~640 cm^−1^ is strongly contributed by vibrations of the C–S bond in oxidized cysteine (cysteic acid, C–SO_3_H) and oxidized methionine (methionine sulfoxide, C–SO–CH_3_). On the other hand, the band at ~655 cm^−1^ is assigned to C–S stretching in “free” bonds (i.e., C–SH) and in “bonded” form (i.e., including both C–S–S–C and C–S–CH_3_) [83,84,85]. Cysteic acid and methionine sulfoxide correspond to the oxidized structures of cysteine and methionine, which are found to generally form during protein oxidation in stressed cells [86,87,88]. Therefore, the observed increase in relative intensity of the 640/655 cm^−1^ doublet should reflect oxidation. On the other hand, in a highly alkaline environment, the flip in relative intensity between these two bands (i.e., the increase in relative intensity of the 655 cm^−1^ band with respect to the one at 635 cm^−1^) could be interpreted as a confirmation of disulfide bond formation. There are also additional spectroscopic fingerprints for the oxidation of S-containing amino acid residues. First, two new bands appeared at 1043 cm^−1^ (cf. arrowed asterisks in Figure 3) and 1623 cm^−1^ (cf. arrowed asterisks in Figure 3 and Figure 8b–d), respectively. These two signals can be assigned to S=O stretching and NH_3_^+^ in-plane bending, respectively; both vibrations appeared as shoulder bands but were clearly missing or very weak in the unexposed virion spectrum. According to Torreggiani et al. [38], the appearance of S=O and NH_3_^+^ reveals the formation of methionine sulfoxide, which is a post-translational product of methionine forming as a consequence of exposure to both non-radical and free radical species. Second, one could note a significant enhancement of two bands from –COO^−^ terminal bonds, whose symmetric and asymmetric stretching vibrations display at 1414 and 1590 cm^−1^, respectively (cf. Figure 3 and Figure 5b–d). In Section 3.1, we substantiated both non-radical and radical reactions that could have caused methionine oxidation at the surface of Si_3_N_4_ bioceramics. Figure 9a shows a schematic draft of the proposed interaction between methionine residues contained in the envelope proteins of HSV-1 virions and deprotonated silanols formed in the highly alkaline environment at the Si_3_N_4_ solid surface. In (b), a draft is offered for the HSV-1/Si_3_N_4_ interaction during hydrolytic reaction in a highly alkaline environment. The striking differences in the virions’ Raman spectra before and after Si_3_N_4_ exposure point to a sequence of events starting from a prompt adsorption of methionine (and cysteine) residues on the Si_3_N_4_ surface, with subsequent fundamental modifications of both the thioether group and terminal structure. The most probable scenario (Figure 9a) behind the electrostatic attraction between Si_3_N_4_ surface and envelope proteins involves deprotonated silanol groups at the surface of Si_3_N_4_ strongly attracting the C-COOH terminus of methionine and cysteine residues. Deprived of a C–S bond on the CH_3_ side, methionine residues undergo thioether cleavage under protonated amino group action, which acts as a hydrogen bond donor and forms strong hydrogen bonds with hydrogen bond acceptor silylamine sites on the Si_3_N_4_ surface [89]. The strong propensity of secondary silylamines to bond with carbon then induces a direct link with the methionine methyl group (CH_3_) to form a quaternary amine with a positive charge. Once the virion detaches from the Si_3_N_4_ particle, the thioether group of the methionine residue cleaves while its C-COOH group deprotonates, thus transforming the methionine residue into a zwitterionic homocysteine form with potentially low or null efficiency in entering the host cells.

#### 3.2.3. Damage and Fragmentation of the DNA Structure

The main effects that ammonia induces on DNA include deamination, strand breaking, cross-linking, and alkylation [79,90,91]. Deamination is the removal of an amino group (NH_2_) from nucleotides within DNA. This can lead to the conversion of cytosine to uracil or adenine to hypoxanthine. Deamination can thus result in the formation of DNA base mismatches, potentially leading to mutations during DNA replication or impaired DNA functioning. Strand breaking can be caused by both direct chemical interaction with NH_3_ molecules and NH_3_-induced oxidative stress. The latter, in turn, leads to the generation of ROS, which can directly damage DNA residues. In DNA cross-linking, DNA strands become covalently linked together. This can occur through the formation of adducts between ammonia-derived RNS and DNA bases or through the generation of reactive species that can cross-link DNA strands. On the other hand, alkylation directly impacts the structural stability of DNA functional groups. Being a nucleophilic molecule, NH_3_ can react with the DNA phosphodiester bonds as well as with guanine and adenine residues, thus breaking backbone and ring structures, respectively. The most striking effects that we observed in analyzing and comparing the Raman spectra of HSV-1 virions before and after exposure to Si_3_N_4_ powder were, as follows: (i) a reduction to ~1/4 and ~1/2 of the DNA phosphodiester bands representing symmetric and antisymmetric O–P–O stretching at 786 and 831 cm^−1^, respectively (cf. Figure 3); and (ii) the almost complete disappearance of ring C–N stretching signals in adenine and guanine residues (at 1336 and 1485 cm^−1^, respectively). Conversely, a lesser and null impact was found on the ring-breathing signals of cytosine and thymine residues, respectively (at 1528 and 1374 cm^−1^, respectively; cf. Figure 7a,b), which demonstrates their higher stability in the presence of NH_3_ and related nitrogen radicals. This scenario precisely matches what has been reported in previous literature [90,91]. Figure 9c,d shows a schematic diagram of the chemical reactions leading to DNA backbone cleavage and a draft of damaged HSV-1 undergoing a structural order → disorder transition due to the presence of ammonia, respectively. As seen in Figure 9c, backbone damage starts with deprotonation at the 2′-hydroxyl group by NH_3_, a process that destabilizes the ribose ring chain with the formation of a transient pentaphosphate and finally leads to bond cleavage upon further interaction with acidic NH_4_^+^ ions [92].

Unlike DNA backbone cleavage, which is caused by direct interaction with NH_3_, disruption of guanine and adenine rings, as hinted by the disappearance of their respective ring C–N stretching signals from the Raman spectra of Si_3_N_4_-exposed virions, can only occur through the strong oxidizing effect operated by O=N–O–O^−^ and not directly through interaction with NH_3_. This observation confirms the occurrence of a cascading reaction ending with the formation of toxic concentrations of highly reactive nitrogen radicals [74,93]. Additional damage to the DNA structure could also occur by deamination and alkylation, which could actually be precursors to ring damage in adenine and cytosine. However, the extent was difficult to assess by Raman spectroscopy due to overlap with amino acid residues not belonging to DNA. All the described effects have a strong impact on DNA stability, affecting its replication and transcription processes. Finally, note that the Raman data on Si_3_N_4_-treated HSV-1 perfectly matches the outputs of immunochemistry assessments shown in Figure 1 and Figure 2.

#### 3.2.4. Destabilization of the Protein Secondary Structure

Raman data reported in Figure 8 have shown a significant alteration of the protein secondary structure in Si_3_N_4_-exposed virions as compared to the control ones. The observed trend is consistent with an increase in the pH of the virions’ environment [94]. The observed one-order-of-magnitude increase in the β-sheet fraction, mainly at the expense of the original fraction of α-helix (cf. Figure 8b,c), points to a destabilization process of the α-helix structure as triggered by the disruption of its hydrogen bonds in a highly alkaline environment (Figure 9e) [95]. Changes in pH can influence the protonation state of amino acid residues in glycoproteins, potentially leading to conformational changes in the protein structure and affecting the stability and function of the glycoprotein. Disruption of the native folded structure of the protein (referred to as denaturation) leads to the loss of its functional properties. Ammonia-induced denaturation likely occurs due to disruptions in the hydrophobic interactions and hydrogen bonding within the protein structure. Denatured proteins may then aggregate, forming insoluble aggregates. Viral glycoproteins participate in protein-protein interactions, including receptor-ligand interactions or interactions with cellular components. Ammonia exposure potentially disrupts these interactions, thereby affecting the function and stability of glycoproteins. Such dramatic structural modifications ultimately lead to a loss of glycosylation sites in envelope glycoproteins. The above interactions impact the stability, function, and conformation of envelope glycoproteins, potentially affecting their role in viral attachment, entry, and other cellular processes (Figure 9f). It should also be noted that, not only is RNS damage to the structure of the lipid envelope incorporating the glycoproteins possible [96], the nucleophilic nitrogen of NH_3_ molecules liberated from the Si_3_N_4_ surface could also directly react with certain functional groups present in glycoproteins. NH_3_ participates in alkylation reactions, where it can covalently bind to certain amino acid residues, such as cysteine or lysine, leading to the formation of stable adducts [97]. Additionally, ammonia can react with the carboxyl groups of aspartic acid or glutamic acid residues, resulting in the formation of amide bonds. Turner et al. [98] proposed an assay that allows the measurement of HSV-1-induced membrane fusion in the absence of HSV-1 infection. Their results showed that HSV-1 envelope glycoproteins are necessary and sufficient for the viral fusion process. In summary, the chemical interactions of ammonia with glycoproteins induce severe structural changes, post-translational modifications, denaturation, and potential aggregation, which can fully justify the observed inactivation of HSV-1 virions.

### 3.3. The Destabilizing Effect of ZrO_2_ Powder on HSV-1 Virions

Experiments with ZrO_2_ particles were initially conceived as a negative control for the antiviral behavior of Si_3_N_4_ particles. However, these experiments revealed a small but not completely null antiviral effect for ZrO_2_ ceramic particles (virus reduction rates ~68% and 96% at 1 min exposure for virions in supernatant and on particles, respectively; cf. Figure 1c). While some mechanistic effect could be invoked to explain the data of virions on particles, it remains to be explained why a virus reduction effect could also be observed in virions from the supernatant.

When ZrO_2_ is exposed to water, hydroxyl groups (OH^−^) from the water molecules can adsorb onto the solid surface, creating an OH^−^-covered surface layer. However, only a slight fraction of Zr^4+^ ions can be released into water. The concentration of Zr^4+^ ions in solution depends on the pH of the water; the higher the pH level, the higher the concentration of Zr^4+^ ions in solution. Oxygen molecules dissolved in water usually adsorb onto the surface of ZrO_2_, reacting with Zr^4+^ ions in the presence of hydroxyl groups, forming a stable zirconia oxide layer that acts as a barrier, preventing further dissolution [99]. According to the above understanding, zirconia is expected to be stable in water and to have no significant interaction with virions at physiological pH. Therefore, unlike other metal oxides (i.e., ZnO, TiO_2_, Fe_2_O_3_, and CuO), which were reported to possess superior (intrinsic) antimicrobial properties [100,101,102], ZrO_2_ usually exhibits negligible virucidal properties unless purposely enhanced with biologically active molecules [103,104,105,106,107].

In a biological environment, however, the interaction between zirconia and cells, bacteria, or viruses can potentially lead to the formation of ROS at the biological interface. The specific mechanisms and extent of oxygen radical formation relate either to byproducts of metabolic activity or to electron transfer processes. ZrO_2_ could indeed exhibit electron transfer capabilities through redox reactions involving enzymes or other biomolecules, which ultimately lead to ROS generation. For instance, interactions between zirconia and cellular oxidases can result in ROS production [108], an argument that Asadpour et al. [109] adopted to explain a dose-dependent effect and an increased cytotoxicity of ZrO_2_ particles on cell lines. ROS include superoxide radicals (•O_2_^−^), hydrogen peroxide (H_2_O_2_), and hydroxyl radicals (•OH), which are highly reactive and can induce oxidative stress within virions. Damage to the virions’ structure includes oxidative modifications of viral proteins, lipids, and nucleic acids [110,111,112]. Such modifications can disrupt the virions’ structure and functions, potentially affecting their ability to infect host cells and/or replicate. DNA damage includes single-strand breaks, double-strand breaks, and base modifications. Finally, the ROS can directly target and impair viral enzymes essential for viral replication, such as viral polymerases or proteases, thus hindering the viral life cycle and reducing viral replication [113]. Raman experiments on ZrO_2_-exposed virions have indeed revealed this kind of damage, although to a minor extent as compared to Si_3_N_4_-exposed virions (cf. Figure 6, Figure 7 and Figure 8 and related discussion in Section 3.2). However, it is important to note that herpes viruses have developed various strategies to counteract oxidative stress and maintain their infectivity. They possess their own antioxidant defense mechanisms, including enzymes such as superoxide dismutase and catalase, to mitigate the damaging effects of ROS [114]. Additionally, herpes viruses can manipulate host cell antioxidant pathways to create a more favorable environment for their replication. These could be the main reasons why the ROS-driven virucidal effect of ZrO_2_ displays a much lower level than the RNS-driven one induced by Si_3_N_4_. Overall, while ROS could temporarily induce damage to the herpes virus, the virus’s antioxidant defenses and its ability to adapt to oxidative stress will support its persistence and ability to cause recurrent infections.

As an additional consideration, it should be noted that inactivation-upon-contact is related to the probability that a virion meets a ceramic particle in suspension. While the inactivation effect of ammonia could be considered as occurring instantaneously (i.e., by straight penetration of NH_3_ molecules inside the intraviral domain), the contact probability depends on time, ceramic particle volume fraction, and electrical attraction between virions and particles. The isoelectric point of HSV-1 is 4.9 [115], which is close to that of Si_3_N_4_ (~4.4) [21,22]; therefore, the contribution of electrical attraction (i.e., the “catching” ability) should be considered to be low. Accordingly, the contact probability could only be enhanced by increasing the particle volume fraction or time in suspension. The choice of a 15 vol.% fraction of ceramic particles in suspension, which is based on previously reported antiviral assessments at a series of different volume fractions and times [22], represents a compromise between having a negligible effect in terms of viscosity of the aqueous suspension and yet having a high enough virion/particle contact probability within 1 min. In the case of 3Y-ZrO_2_ particles (isoelectric point: 6.6~7.2) [116], the electrical attraction is much higher, but the effect of oxygen radicals is much weaker than ammonia (since the effect of oxygen radicals is confined to the virions’ surface). A longer time in suspension (i.e., 10 min) was set to give time for the inactivation mechanism to fully operate, but the effect of oxygen radicals was still conspicuously weak. In summary, if a “catch and kill” mechanism is envisaged for viral inactivation [20], Si_3_N_4_ and ZrO_2_ powders exploit higher “killing” and “catching” abilities, respectively, against HSV-1. However, the poisoning effect of ammonia from Si_3_N_4_ surface hydrolysis appears to be the most effective mechanism for the inactivation of HSV-1.

### 3.4. The Direct and Matrix-Based Use of Si_3_N_4_ against Herpes Viruses

A preponderant fraction of the adult population around the world carries the Herpes simplex virus Type-1, with a smaller percentage infected with Type-2 [117]. In particular, herpes labialis is mainly caused by HSV-1; type-2 viruses can also lead to primary orofacial infections, but they rarely cause relapses [118]. HSV-1 orofacial infections are usually harmless and self-healing in immunocompetent patients. Even in such patients, they involve recurrent rashes of the skin and mucous membranes (in particular, the lips) with erythema and blisters that are accompanied by burning pain. The intrinsic capacity of Herpesviruses to survive (either in lytic phase during replication or in long-term latency when functionally dormant in ganglia) and their contagiousness toward yet uninfected and immunocompromised individuals (i.e., affected by HIV or undergoing chemotherapy) [119] call for effective methods of both treatment and prevention.

Treatments for Herpesviruses include oral antiviral medications and topical (antiviral or indifferent) creams. Oral administration of acyclovir or its prodrug, valacyclovir, is nowadays the most commonly prescribed treatment [120]. However, these oral medications often show inefficient viral suppression, a circumstance mainly due to their poor oral bioavailability [120,121,122] and short (2.5~3.3 h) in vivo plasma elimination half-life [123,124]. Antiviral creams incorporating acyclovir have also been widely investigated [125,126,127,128,129,130,131,132,133,134,135]. Even when applied immediately after the first appearance of prodromal symptoms, these creams were found to be barely capable of reducing the duration or severity of pain, although a reduction in recovery time has been seen in some early studies [125,126]. Indifferent creams, which typically contain small fractions of either zinc oxide or zinc sulfate [136,137], were found to shorten recovery time by ~20% in individuals who received treatment promptly upon the appearance of symptoms. To their disadvantage, burning sensations, itchiness, and dryness were side effects noted for zinc oxide/glycerin cream and zinc sulfate gel, respectively [136]. In a recently proposed approach, Giannasca et al. [138] suggested a new approach to local distribution of acyclovir via sustained drug release through the incorporation of the antiviral agent into a polymeric matrix with the shape of an antiherpetic ring. This approach showed potential improvement with respect to the efficacy of the orally administered acyclovir drug and aimed at abrogating the two main shortcomings of oral dosing, namely, poor absorption by the gut and a rapid drop in plasma concentration (cf. above).

In the described therapeutic scenario for oral administration and in view of the above-mentioned disadvantages (with the possibility of poor patient compliance) for the presently available topical remedies, the present findings of full and instantaneous inactivation of HSV-1 using a micrometric powder with a minor fraction of Si_3_N_4_ could provide a valid alternative topical antiviral treatment. Applications could include the use of small fractional dispersions of Si_3_N_4_ powder into indifferent creams, gels, and oral rinses (in substitution of zinc oxide and zinc sulfate) or its local exploitation via sustained hydrolytic release in polymeric matrix-based antiherpetic rings or mouthpieces. This latter approach is similar to that pursued by Giannasca et al. [138], but it employs a solid-state antiviral compound rather than an absorbed drug incorporated in the polymeric matrix. Among the expected advantages, one could foresee high local effectiveness exactly at the location where herpes symptoms appear, easy maintenance, and strong prophylaxis with none of the shortcomings associated with oral uptake (e.g., poor absorption and renal metabolism of the drug before its reaching the target tissue). Note also that incorporation of small fractions of micrometric Si_3_N_4_ powder in polymethyl methacrylate matrix is an approach already successfully applied against Candida pathogens for application in dentures [139] and in polyetheretherketone matrix against bacterial infections for application in spinal fusion implants [140]. Si_3_N_4_-based antiviral medications or devices are completely nontoxic in human cell culture [20,63] and can suppress primary HSV-1 replication with continuity in time and constant efficacy (cf. Section 3.1). We hope that the present data will trigger further investigations into antiviral Si_3_N_4_ applications and form the basis for novel therapeutic interventions against herpes and other persistent viral infections.

## 4. Materials and Methods

### 4.1. Virus Sample and Cell Infection Procedure

Experiments with the Human herpesvirus 1 (HHV-1, also referred to as Herpes simplex virus type 1 or HSV-1) Amakata strain were conducted in a Biosafety Level 2 (BSL-2) biocontainment facility according to BSL-2 work practices. The investigated herpes virus has long been maintained in our laboratory [24,25]. Purchased ATCC CCL-81 Vero cells were cultured in growth medium (GM), Dulbecco’s modified Eagle’s minimum essential medium (DMEM) (Nacalai Tesque, Inc., Kyoto, Japan), supplemented with penicillin (100 units/mL), streptomycin (100 lg/mL), and 5% fetal bovine serum. The maintenance solution was DMEM supplemented with penicillin, streptomycin, and 1% fetal bovine serum. The Vero cells were seeded in T-175 flasks (Falcon) and grown in GM upon maintaining them at 37 °C in an atmosphere of 5% CO_2_.

Vero cells were infected with HSV-1 at a multiplicity of infection (MOI) of 0.1 and incubated at 37 °C in a 5% CO_2_ incubator for 2 days. The culture supernatant was then harvested and centrifuged at 3000 rpm for 20 min. The clarified supernatant was filtered, aliquoted, and then stored at −80 °C until use. A virus concentration corresponding to the order of 10^9^ TCID_50_ was applied to each glass bottom (an area of 38.5 mm^2^; 7 mm in diameter).

### 4.2. Ceramic Powders, Inactivation Protocol, and Virus Titration

The Si_3_N_4_ powder used in antiviral experiments had an average particle size of 1.6 ± 0.4 µm, as measured by means of a particle size analyzer (ELSZ-1000, Otsuka Electronics, Osaka, Japan). This powder was obtained upon mechanical grinding and successive filtration of a sintered ß-Si_3_N_4_ body having a nominal composition of 90 wt.% ß-Si_3_N_4_, 6 wt.% yttrium oxide (Y_2_O_3_), and 4 wt.% aluminum oxide (Al_2_O_3_). The raw material used to produce the ceramic sintered body was the high-purity powder UBE E-10 (Ube Industries, Ube, Japan). The sintered body was densified in a nitrogen atmosphere at 1750 °C for 3 h. Similar to the case of the Si_3_N_4_ powder investigated in Ref. [22], X-ray photoelectron spectroscopy (JPS-9010 MC; JEOL Ltd., Tokyo, Japan) performed with an X-ray source of monochromatic MgKα (output 10 kV, 10 mA) revealed a Si_2p_ edge composed of four distinct sub-bands, which could be assigned to N-Si-N, N-Si-O, N-Si-O_x_, and O-Si-O. Upon immersion in aqueous solution at neutral pH, a change in the relative intensities of XPS sub-band components revealed a decrease in the N-Si-N bonds counterbalanced by an increase in the N-Si-O bond population. This variation was interpreted as an experimental proof of surface hydrolysis of the Si_3_N_4_ powder with subsequent elution of ammonium/ammonia ions [63].

The ZrO_2_ powder used in antiviral experiments was also obtained upon mechanical grinding (and successive filtering) of a sample pre-sintered (at 970 °C for 1 h) and then densified (at 1500 °C for 2 h) from a high-purity ZrO_2_ powder containing 3 mol% Y_2_O_3_ (Zpex; Tosoh, Tokyo, Japan). The grinding and filtering procedures were the same as those employed for the Si_3_N_4_ powder. The obtained particle size, 1.3 ± 0.7 µm, was comparable with that of the Si_3_N_4_ powder. After preparation, both powders were heat-sterilized at 180 °C for 2 h before being used in antiviral experiments.

The ceramic powders were adjusted to 15 vol.% with PBS (Nacalai Tesque, Inc., Kyoto, Japan). Twenty microliters (2 × 10^5^ TCID_50_) of virus solution were added to 1 mL of sample solutions, then mixed for 1 min in a rotating machine at room temperature, followed by centrifugation at 12,000 rpm at 4 °C for 2 min. The experiment was performed in triplicate for each sample (*n* = 3). Each supernatant was subjected to virus infectivity titer measurement and a Real-Time Polymerase Chain Reaction (RT-PCR) test. The viral titer was assayed by a Median tissue culture infectious dose (TCID_50_) (or plaque-forming method) using Vero cells (as described above). TCID_50_ was calculated by the standardized Reed-Muench method.

### 4.3. Polymerase Chain Reaction Procedures

DNA extraction from viruses was performed on a virus solution mixed 1:1 with a 2× TNE Buffer and allowed to stand at 37 °C for 1~2 h (final concentration of TNE Buffer: 50 mM Tris-HCl pH 8.0, 0.5% SDS, 25 mM EDTA, 100 mM NaCl, 200 µg/mL Proteinase K). Deproteinization was performed by Phenol/Chloroform/Isoamyl alcohol treatment and then ethanol precipitation; 100 µL of TE was added to obtain a DNA solution. Forward: 5’-ctgcttagcgccaaggtca-3’ and Reverse: 5’-tgggaacttgggtgtagttggt-3’ HSV-1 primers and probes: 5’-FAMcgacatggtcatgcgcaagcgBHQ-1-3’ were used in performing the quantitative polymerase chain reaction (qPCR) of HSV-1 DNA. The DNA was finally subjected to quantitative real-time PCR using a StepOnePlus Real-Time PCR System (Thermo Fisher Scientific Inc., Waltham, MA, USA).

### 4.4. Raman Samples and Spectroscopic Procedures

A viral solution was pelleted by ultracentrifugation at 28,000 rpm for 2.5 h using an Ultracentrifuge Optima XL-100K rotor SW28 (Beckman Coulter Inc., Brea, CA, USA). The virus was then re-suspended in a small amount of PBS and mixed with micrometric Si_3_N_4_ or ZrO_2_ powder, as described above, for the inactivation protocol. The centrifuged virions were then placed on a glass bottom dish (MatTek Life Sciences, Ashland, MA, USA), air-dried, and fixed in 4% paraformaldehyde at room temperature for 10 min. After virus fixation, samples were washed twice with PBS and then twice again with DW. Virus samples were dried in the air and stored at 4 °C until Raman analyses. A control virus sample was obtained by exactly the same procedure, but without the addition of ceramic particles.

Raman experiments were conducted by employing a specially designed spectrometer (LabRAM HR800, Horiba/Jobin-Yvon, Kyoto, Japan) set in confocal mode. A holographic notch filter simultaneously provided high-efficiency and high-resolution spectral acquisitions. The wavelength of the incoming laser was 785 nm, with the laser source operating at a laser power of 70 mW. The Raman scattered light was analyzed with a double monochromator connected with an air-cooled charge-coupled device (CCD) detector (Andor DV420-OE322; 1024 × 256 pixels); the grating used in the spectrometer had a resolution of 1800 gr/mm. The spectral resolution was ~1 cm^−1^. To eliminate noise, the acquisition time of a single spectrum was typically 20 s for three consecutive acquisitions at the same location. The laser spot was ~2 μm in width as it was focused on the sample through a 50× optical lens. Sets of ten spectra were collected at different locations on each sample (over areas of ~2 mm^2^) and averaged in order to obtain an average spectrum used throughout the analyses. Raman spectra were subjected to baseline subtraction according to the asymmetric least squares method [26]. The average spectra were deconvoluted into a series of Lorentzian-Gaussian sub-bands using commercial software (LabSpec 4.02, Horiba/Jobin-Yvon, Kyoto, Japan). In performing this deconvolutive procedure, a machine-learning approach was applied, which employed an in-house-constructed automatic solver described in previous studies [27,28].

### 4.5. Statistical Analyses

Data are presented as the mean value ± standard deviation of three independent experiments (*n* = 3). The statistical difference between virions exposed to ceramic powders and unexposed ones (control samples) was analyzed by computing mean values and standard deviations. Their statistical validity was evaluated by applying the ANOVA analysis of variance. Statistically significant *p*-values are indicated in the inset of each plot and labeled with asterisks.

## 5. Conclusions

The present study suggests the suitability of Si_3_N_4_ micrometric powder in an aqueous environment as a possible treatment or adjuvant therapy for HSV-1 infections. Minor fractions of micrometric Si_3_N_4_ particles could be loaded into antiviral creams and gels or suspended in oral rinses to exploit an extremely efficient and instantaneous viral reduction effect. Moreover, incorporation of a minor fraction of micrometric Si_3_N_4_ particles into a polymeric matrix could be used for the fabrication of antiherpetic devices, effectively fighting viral reactivation and enabling high local effectiveness exactly at the location where infection would appear. Raman spectroscopic analyses performed on HSV-1 exposed for only 1 min to an aqueous solution containing 15 vol.% Si_3_N_4_ particles (in comparison with control virions) revealed extensive structural damage, including oxidation of sulfur-containing amino acid residues, DNA ring disruption and fragmentation, and destabilization of envelope glycoprotein secondary structure. Assessments for longer periods (up to 10 min) in aqueous solution did not significantly change the outputs for the observed antiviral behavior of Si_3_N_4_, which was already >98% after 1 min of exposure. It is likely that other nitrides undergoing hydrolysis, such as aluminum nitride, could be analogously employed for HSV-1 treatment; nevertheless, some peculiar characteristics of the Si cation, such as its biocompatibility with respect to human cells and full resorbability in a biological environment with no permanent contamination of human tissues, render the Si_3_N_4_ bioceramic a unique solid-state antiviral agent.

Future studies should address the antiviral effectiveness (and the related antiviral mechanisms) of Si_3_N_4_ against different types of herpes viruses in a more relevant milieu than simple in vitro testing.

## Data Availability

Data available on request from the authors.
